# Green Synthesis and Characterization of Novel Silver Nanoparticles Using *Achillea maritima* subsp. *maritima* Aqueous Extract: Antioxidant and Antidiabetic Potential and Effect on Virulence Mechanisms of Bacterial and Fungal Pathogens

**DOI:** 10.3390/nano13131964

**Published:** 2023-06-28

**Authors:** Badiaa Essghaier, Hédia Hannachi, Rihem Nouir, Filomena Mottola, Lucia Rocco

**Affiliations:** 1 Laboratory of Biochemistry and Biotechnology LR01ES05, Department of Biology, Faculty of Sciences of Tunis, University Tunis El-Manar, Tunis 2092, Tunisia; 2 Laboratory of Vegetable Productivity and Environmental Constraint LR18ES04, Department of Biology, Faculty of Sciences, University Tunis El-Manar II, Tunis 2092, Tunisia; hedia.hannachi@fst.utm.tn; 3 Laboratoire de Spectroscopie Atomique, Moléculaire et Applications (LSAMA), Faculty of Sciences, University Tunis El-Manar, Tunis 2092, Tunisia; rihem.nouir@gmail.com; 4 Higher Institute of Medical Technologies of Tunis, University Tunis El-Manar, Tunis 2092, Tunisia; 5 Department of Environmental, Biological and Pharmaceutical Sciences and Technologies (DiSTABiF), University of Campania “L. Vanvitelli”, 81100 Caserta, Italy; filomena.mottola@unicampania.it

**Keywords:** silver nanoparticles, *Achillea maritima*, antioxidant, antibacterial, antifungal, antidiabetic, mechanisms of action

## Abstract

Novel silver nanoparticles were synthesized based on a simple and non-toxic method by applying the green synthesis technique, using, for the first time, the aqueous extract of an extremophile plant belonging to the *Achillea maritima* subsp. *maritima* species. AgNP characterization was performed via UV-Visible, front-face fluorescence spectroscopy, and FTIR and XRD analyses. AgNP formation was immediately confirmed by a color change from yellow to brown and by a surface plasmon resonance peak using UV-Vis spectroscopy at 420 nm. The biosynthesized AgNPs were spherical in shape with a size ranging from approximatively 14.13 to 21.26 nm. The presented silver nanoparticles exhibited strong antioxidant activity following a DPPH assay compared to ascorbic acid, with IC50 values of about 0.089 µg/mL and 22.54 µg/mL, respectively. The AgNPs showed higher antidiabetic capacities than acarbose, by inhibiting both alpha amylase and alpha glucosidase. The silver nanoparticles could affect various bacterial mechanisms of virulence, such as EPS production, biofilm formation and DNA damage. The silver nanoparticles showed no lysozyme activity on the cell walls of Gram-positive bacteria. The AgNPs also had a strong inhibitory effect on the *Candida albicans* virulence factor (extracellular enzymes, biofilm formation). The microscopic observation showed abnormal morphogenesis and agglomeration of *Candida albicans* exposed to AgNPs. The AgNPs showed no cytotoxic effect on human cells in an MTT assay. The use of novel silver nanoparticles is encouraged in the formulation of natural antimicrobial and antidiabetic agents.

## 1. Introduction

Nanoscience has gained the attention of researchers as nanoparticles and nanomaterials can have different uses, including in the pharmaceutical, biomedical, food technology and cosmetic fields [1]. Silver nanoparticles (AgNPs) are more important in biological applications owing their significant properties, such as their unique physicochemical properties and biosafety. In fact, AgNPs are commonly used as antimicrobial, anticancer and antidiabetic agents and as biosensors [2]. The green synthesis of metallic nanoparticles is easier, less costly and more eco-friendly compared to other chemical and physical methods [3].

Extremophiles are used for the green synthesis of nanomaterials due to their high quality, and the ecofriendly production of metallic nanoparticles has garnered great industrial interest [4]. In the strategy for improving the activity and sensitivity of natural silver nanoparticles, we continue our work to search for novel nanoparticles using extremophile plant extracts [5,6].

The *Achillea* genus belongs to the well-known medicinal plant from the Asteraceae family. It contains over 100 species of wild-growing plants. In addition, people around the world use *Achillea* in the treatment of various diseases [7]. In turkey, *Achillea* species are used as natural resources for treating gastrointestinal complaints, as an anti-inflammatory, for wound healing and as an anti-diuretic [8]. On the other hand, *Achillea* species exhibit various biological activities such as angiogenic [9], antifungal [10], antibacterial [11], antioxidant [12], antimicrobial [13] and antidiarrheal [14] activities.

Cottonweed (*Achillea maritima* L.) is xerophyte, psammophyte, medicinal herbaceous species found on sea dunes. Cottonweed is a species with optimum Mediterranean distribution found along the coasts of the Mediterranean and the Atlantic coasts of Europe. In Italy, it is present in all central, southern and island regions. Regarding the *Achillea* genus, rare species, such as *Achillea biederteinii* [15], *Achillea wilhelmssi* CKoch (Aw) [16] and *Achillea millefolium* [17], are used for the biosynthesis of silver nanoparticles. To our knowledge, there are no reports about the green synthesis of silver nanoparticles from *Achillea maritima*. The current pioneering work focuses on the green synthesis of AgNPs from *Achillea maritima* growing on the sea dunes on the northeastern Mediterranean coast of Tunisia. There are also no reports on the antimicrobial activity of *Achillea maritima* aqueous extract, unlike various *Achillea* species, like *Achillea millefolium* [18], *Achillea setacea* [19] and *Achillea santolina* [8], whose essential oils have previously been studied as antimicrobial agents.

Antibiotic and antimicrobial resistance (AMR) are a global problem posing human and animal health risks; this problem leads to higher medical costs and increased mortality. Therefore, several novel alternatives have been developed to try to combat this resistance, including silver nanoparticles [20]. With the urgent need for new antibacterial agents, due to their synergistic effects against Gram-positive and Gram-negative bacteria as well as against multi-resistant bacteria and in combination with antibiotics, AgNPs are a suitable alternative to the conventional antibiotic [21].

Diabetes mellitus is a severe complex metabolic disorder. Synthetic antidiabetic compounds, such as acarbose and miglitol, show serious side effects such as swelling, stomach pain and diarrhea; therefore, we need more effective and safe antidiabetic agents for the treatment of diabetics [22].

On the other hand, the efficiency of green-synthesized AgNPs in inhibiting alpha amylase and alpha glucosidase enzymes suggests their potential for treating diabetes mellitus [23]. In fact, many silver nanoparticles have shown antidiabetic activities, e.g., [6,24,25]. Here, we report on new silver nanoparticles and evaluate their antidiabetic activities. Furthermore, owing to their different phytochemicals that act as reducing and capping agents, necessary for the green synthesis of silver nanoparticles, they play an important role in biomedicine as a promising alternative for the treatment of a wide range of diseases, including cancers [5,16,26].

In the literature, some reports conduct fluorescence analysis of silver nanoparticles. Here, we propose the use of front-face fluorescence spectroscopy (FFFS) to analyze and identify our biosynthesized silver nanoparticles [27,28,29,30,31]. The fluorescence of silver nanoparticles can be used to describe their surfaces/interfaces as previously described [30]. A particularly sensitive technique that can find very minute concentrations of silver nanoparticles is UV-Visible fluorescence. For instance, this technique can be used to find biosynthesized silver nanoparticles using bacterial cultures or plant extracts. Unchanged silver nanoparticles are not necessary for the non-destructive detection technique known as UV-Visible fluorescence. As a result, this technique enables the study of silver nanoparticles in their natural condition, which can be crucial for comprehending how they behave in intricate biological contexts [29,30,31].

In light of the above data, the present study, for the first time, aims to conduct green synthesis and characterization of new silver nanoparticles using *Achillea maritima* aqueous extract. Secondly, we investigate their biological activities by evaluating their antioxidant, antidiabetic, antibacterial, anti-fungal potential, in addition to evaluating their mechanism of action.

## 2. Materials and Methods

### 2.1. Preparation of Plant Extract and Biosynthesis of Silver Nanoparticles

**Figure 1:**
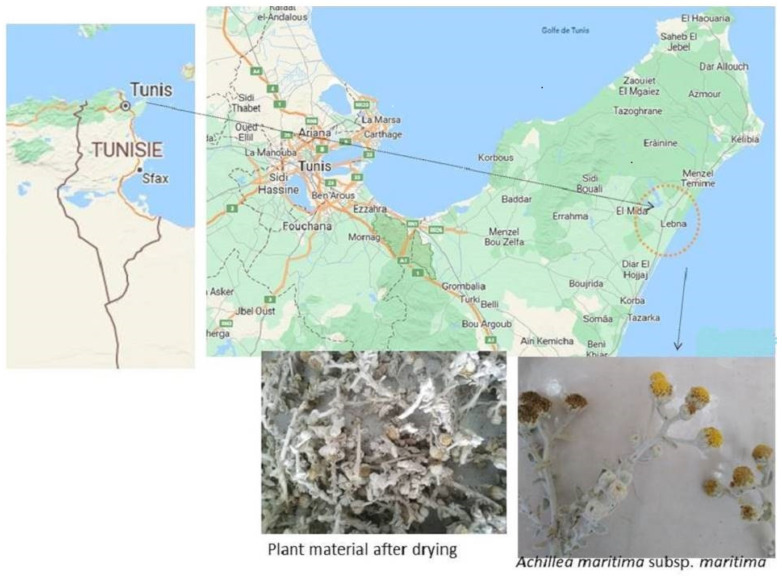
Tunisia map showing the location of the sample xerophyte cottonweed species *Achillea maritima* L. used for the green synthesis of silver nanoparticles.

*Achillea maritima* was identified by a specialist and deposited at the herbarium laboratory to assign it a voucher number. The plant was collected from the northern coast of Tunisia, and then, washed with distilled water for 15 min, air dried and cut into fine pieces (Figure 1). The plant material was used to make the aqueous extract using 20 g per 100 mL distilled water boiled at 50 °C for 30 min, and left at room temperature overnight. The obtained extract was filtered through Whatman filter paper and conserved at 4 °C until use. To synthesize the silver nanoparticles, 20 mL of 1 mM of silver nitrate solution AgNO_3_ was added to 3 mL of the obtained aqueous extract. The observation of the color change from pale yellow to dark brown indicates the presence of silver nanoparticles. The biosynthesized AgNPs were recuperated after centrifugation at 15,000 rpm at 4 °C for 15 min. The obtained pellet was separated and purified with sterile milliQ water via several centrifugations at 4 °C and 15,000 rpm for 15 min. The dried AgNPs were kept at 4 °C until use [5].

### 2.2. Characterization of Silver Nanoparticles

Different methods were carried out to identify the structure and the size of the new biosynthesized silver nanoparticles, as previously described by Essghaier et al. [3]. UV-Visible absorbance spectroscopy was performed using a 2802 UV/VIS spectrophotometer (UNICO). X-ray diffraction (XRD) measurement was carried out using an X-ray diffractometer (D8 ADVANCE BRUKER) with Cu Ka radiation (λ = 1.5406 Å). Fourier transform infrared spectroscopy (FTIR) analysis was conducted in the range of 400–4500 cm^−1^ using a Varian FTIR640 spectrophotometer.

### 2.3. Experimental Setup of Fluorescence Analysis

For the experiment, the setup employed, based on previous studies [32,33,34,35], bifurcated optical fiber and a UV light electroluminescent diode (LED) at 365 nm with an 8 nm half-width to excite the samples. UV-Vis front-face fluorescence spectroscopy (FFFS) provides fluorescence emission excitation and frontal collection simultaneously. To measure the fluorescence spectrum in the UV-VIS-IR region, the emitted fluorescence light was collected on the same side as the excitation and directed by the optical fiber to a USB-2000 spectrometer with a linear charge-coupled device (CCD) detector. To remove the reflected light from the excitation wavelength that superposed the fluorescence spectra, a high-pass optical filter in the 400 nm range was used. Using SpectraSuite software version 2.0, spectra were acquired between 400 and 800 nm. The same methodology and identical conditions were used for all measurements [32,33,34,35]. We made five recordings for the sample and left a 3 s exposure delay between each recording to allow for stability of the fluorescence signal before each capture. The measured spectra were processed by averaging the five recorded spectra that were acquired for the silver nanoparticles using the software program Igor Pro 6.01, and then, the measured spectra were subjected to deconvolution analysis using Gaussian functions to identify the peak positions. Python was utilized for the spectral deconvolution and treatment [32,33,34,35].

### 2.4. Antioxidant Activity

#### 2.4.1. DPPH Radical Scavenging Activity

A free radical scavenging activity (DPPH) method was used to evaluate the antioxidant activities of the biosynthesized silver nanoparticles. An ethanolic solution of 2,2-diphényl 1-picrylhydrazyle at 200 mM was prepared. The reaction solution contained a 20 µL DPPH solution and 180 µL of AgNPs at different concentrations. After 30 min of incubation in the dark, the absorbance was measured at 515 nm using a microplate spectrophotometer (Bioteck ELx808). A control assay was prepared using solvent without AgNPs, and ascorbic acid was used as a positive standard. The percentage of inhibition was determined using the formula: [(Ac − As)/Ac] × 100, where Ac: absorbance of control; As: absorbance of sample (AgNPs).

The inhibition percentage was measured according different AgNP concentrations to determine the IC50, which represents the concentration of AgNPs able to reduce 50% of the tested radicals [5].

#### 2.4.2. Total Antioxidant Activity (TAA)

The TAA of the AgNPs was determined using the phosphomolybdenum method described previously [3]. The reaction containing 200 µL of AgNPs and 2 mL of the reaction containing 2.3 mL of sulfuric acid (H_2_SO_4_, 0.6 M) and 0.397 g of sodium phosphate (NaHPO_4_) were adjusted with H_2_O at acidic pH. The solution was boiled in a water bath for 90 min. Then, the absorbance was measured at 695 nm, after incubation at room temperature. The blank was prepared under the same conditions. The calibration curve was determined using ascorbic acid at different concentrations. The total antioxidant activity was expressed as mg acid ascorbic equivalent (AAE)/gDM (dry matter) [3].

### 2.5. Antidiabetic Effect

The antidiabetic effect of AgNPs was determined in vitro by evaluating the inhibitory effect of both alpha amylase and alpha glucosidase.

#### 2.5.1. α Amylase Inhibitory Assay

The mix reaction contained 500 µL of AgNP dilution and 500 µL of enzyme solution (alpha amylase at 0.5 mg/mL). After incubation at 25 °C for 10 min, 500 µL of 1% starch solution was added and incubated for 10 min. The reaction was stopped by adding 1 mL of DNS reagent, heated for 5 min and left at room temperature; then, 5 mL of distilled water was added. The absorbance was measured at 540 nm. Acarbose was used as a positive control. The inhibition as a percentage was calculated as previously described [5,36].

#### 2.5.2. α Glucosidase Inhibitory Assay

The inhibitory effect of the silver nanoparticles on yeast alpha glucosidase (Sigma, St. Louis, MO, USA) was determined using the method previously described in [37]. Briefly, 100 µL of the sample (AgNPs or acarbose) at different concentrations (from 10 to 120 µg/mL) was mixed separately with 50 µL of α glucosidase (0.1 U/mL), and incubated at 37 °C for 20 min. Then, 10 µL of p-nitrophenyl-α-d-glucopyranoside (pNPG) was added and incubated for 10 min under the same conditions. To stop the reaction, we added 650 µL of 1 M sodium carbonate Na_2_CO_3_. The absorbance was measured at 405 nm. The percentage of enzyme inhibition was calculated as I (%) = (A405_Control_ − A405_AgNPs_/A405_Control_) × 100.

### 2.6. Cytotoxic Effect on Human Cells

The human colon adenocarcinoma cells line LS174T CL-188 was purchased from ATCC. The MTT assay was applied to determine the cytotoxic effect of the synthesized silver nanoparticles on LS174 CR cancer cells exposed to the silver nanoparticles at concentrations varying from 102.4 µg/mL to 0.4 µg/mL. The percentage of cell viability was measured after 24 h and 48 h of incubation with various AgNP solutions as previously described [5].

### 2.7. Antimicrobial Potential

The antimicrobial activity of silver nanoparticles was assessed against clinical pathogens at a Tunisian public hospital, including Gram-negative bacterial species (*Pseudomonas aeruginosa*, *Salmonella typhi*, *Escherchia coli*) and Gram-positive bacteria *(Staphylococcus aureus)*. Antifungal activity was determined using two fungal species: *Candida albicans* and *Candida tropicalis*. Mueller–Hinton media (BioRad, Mitry-Mory, France) and potato dextrose agar were used for the antibacterial and antifungal assays, respectively.

#### 2.7.1. Agar Well Diffusion Method

To evaluate the antimicrobial potential of silver nanoparticles, the well diffusion technique was used according to Thakur et al. [38]. First, the surface of an agar plate was spread with 0.1 mL of the tested inoculum adjusted to 10^6^ CFU and 10^5^ spores for bacterial and fungal strains, respectively. Then, each well was inoculated with 40 µL of the AgNP dilutions (Supplementary Materials). After incubation, the antimicrobial activity was calculated by measuring the diameter of the inhibition zone. Ceftazidime CAZ30 was used as a positive control. Amphotericin B and Fluconazole 25 were used as conventional fungicides [39].

#### 2.7.2. Minimum Inhibitory Concentration (MIC) and Minimum Bactericidal Concentration

MIC was determined using a sterile 96-well microplate as previously described [40]. To calculate the MBC and MFC, 10 µL from the wells, showing no visible growth that corresponded to the MIC value of the pathogen, was spread on the surface of the agar plate, and incubated at 37 °C for 24 h. The apparition of the colony was verified. All experiments were performed in quadruplicate [38,40].

Okou et al. [41] explain that MBC/MIC or MFC/MIC ratios < 4 indicate a bactericidal and fungicidal effect of the antimicrobial agents. If this ratio is >4, it has a bacteriostatic or fungistatic effect.

#### 2.7.3. In Vitro Silver Nanoparticles’ Effect on Lipopolysaccharides and Bacterial Biofilm

The probable effect of the silver nanoparticles on LPS degradation was assessed in vitro based on the method described in [42]. The reaction contained 1% cells of Gram-negative bacteria, grown in 1 mL LB broth in the presence and the absence of AgNPs (50 µg/mL). After incubation for 18 h at 120 rpm, the culture was centrifuged at 8000 rpm/10 min. The obtained cell pellet was suspended in saline solution (0.9% NaCl); then, an equal volume of 5% phenol was added to the suspension, along with a 5 volume of H_2_SO_4_. The mix was kept for 1 h in the dark, and the absorbance was measured at 490 nm. The effect of the silver nanoparticles on bacterial biofilm formation was examined based on the method of Kim et al. [43].

#### 2.7.4. Silver Nanoparticles’ Effect on DNA Bacterial Genome

The bacterial species were cultured in the presence and the absence of silver nanoparticles at 50 µg/mL. After 24 h incubation at 37 °C, the extraction of the DNA genome was performed using the boiling method, and the DNA was visualized via gel electrophoresis, under UV light [44].

### 2.8. Lysozyme Activity

The lysozyme activity of the silver nanoparticles was determined using the method of Sehimi et al. [45]. The cell wall of Gram-positive bacteria (*Bacillus cereus*, *Staphylococcus aureus* or *Micrococcus luteus*), was suspended in 50 mM phosphate buffer (pH 6.5). The reaction mixture contained *v*/*v* of the bacterial cell wall solution and AgNPs (50 µg/mL), and was incubated at 37 °C for 60 min. The OD was measured at 600 nm, and the decrease in OD with 0.001/mL/min corresponded to 1AU of lysozyme activity [46].

### 2.9. Silver Nanoparticles’ Effect on Candida Species Growth and Virulence

In order to determine the AgNPs’ effect (used at 20 µL of 50 µg/mL) on *Candida* growth and morphogenesis, 100 µL of *Candida* culture (10^5^ spores/mL) was used to inoculate 1 mL of YM medium. After 48 h incubation at 37 °C, under shaking, OD was measured at 600 nm. The percentage of growth inhibition of *Candida* survival was calculated as previously described [6]. The morphology was examined after coloration with blue cotton.

AgNPs’ effects on lipase and proteinase were assessed in egg yolk agar medium and bovine serum albumin medium, respectively, based on the methods of Jin et al. [47] and Mohandas and Ballal [48]. The diameter of colonies and the zone opacity were measured as follows: Pz = colony diameter in mm/zone opacity +colony diameter (mm).

### 2.10. Statistical Analysis

The values are presented as means ± standard error of the means (SEM). Multiple comparison of the means was performed using Student–Newman–Keuls SNK tests at a threshold of 5% (means with the same letters are not significantly different, *n* = 3). The analysis was carried out using XLSTAT software (trial version, www.xlstat.com (accessed on 1 January 2022)).

## 3. Results

### 3.1. Characterization of Silver Nanoparticles

#### 3.1.1. UV-Visible Spectroscopic Analysis

**Figure 2:**
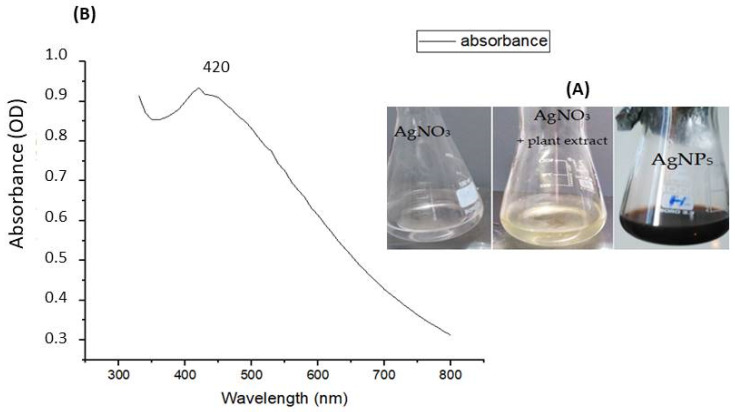
(**A**). Color change of the silver solution, after adding the aqueous extract of *Achillea maritima* L., from light brown (AgNO_3_) to dark brown (AgNPs). (**B**) Surface plasmon resonance (SPR) peak following UV-Vis spectroscopy of the biosynthesized silver nanoparticles.

A color change of *Achillea maritima* L. aqueous extract from pale yellow to dark brown occurred due to the reduction of Ag^+^ ions to Ag^0^, which indicates the formation of silver nanoparticles (Figure 2A). UV visible spectroscopy is a spectrophotometry model commonly used to determine absorbance spectra using a sample solution. The silver nanoparticles’ UV-Vis absorption spectra are illustrated in Figure 2B, showing the surface plasmon resonance (SPR), with maximum absorption at 420 nm. The surface plasmon resonance peak of AgNPs depends on their size. The observation of a single peak proves the spherical shape of the nanoparticles. An average size of 21.26 nm was calculated based on the formula of Amirjani et al. [49].

#### 3.1.2. Fluorescence Analysis

**Figure 3:**
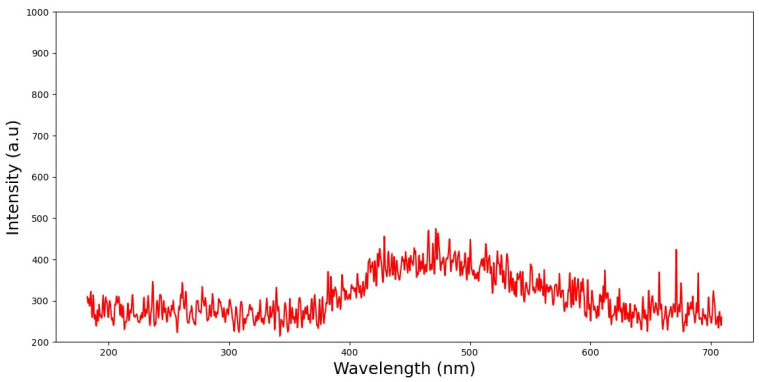
Fluorescence emission spectrum of the biosynthesized silver nanoparticles using Python.

Front-face fluorescence spectroscopy (FFFS) is one approach to estimating the optical property of silver nanoparticles as a photonic material. The chosen methodology entails taking measurements while the samples are illuminated. This is performed via excitation of the samples, and afterwards, an analysis of the fluorescence emission spectra of the biosynthesized AgNPs is performed. Figure 3 shows typical spectra from this analysis that match the AgNPs after being examined in a large number of spectra. The regions corresponding to the shoulder visible in the spectra following excitation at 365 nm have an emission wavelength close to 470 nm. In this work, several fluorescence measurements were carried out in order to analyze and investigate the biosynthesized silver nanoparticles.

**Figure 4:**
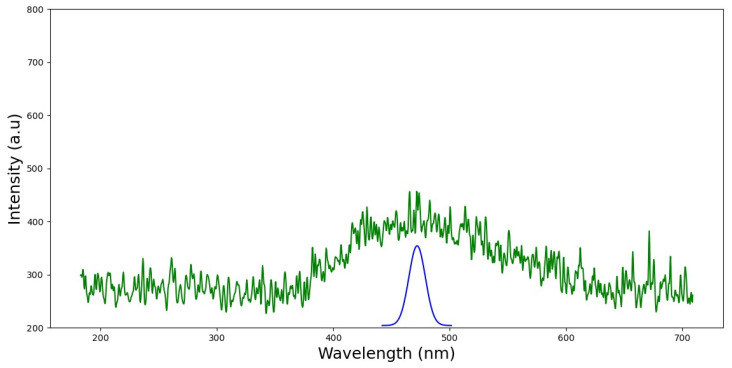
Spectral deconvolution analysis of the silver nanoparticles.

Identification and localization of the AgNPs were made possible through the examination and treatment of all the fluorescence spectra. We sought to refine these results by applying Gaussian functions to deconvolute the collected spectra and identify the peak’s spectral position in order to obtain data about the substances. Figure 4 displays the outcome of this deconvolution method for AgNPs. The wavelength range from 400 to 500 nm is where the noticeable absorption peak is found. The peak of AgNP fluorescence emission is found to be at about 469.829 nm. These findings prove the formation of spherical nanoparticles.

#### 3.1.3. FTIR Analysis

**Figure 5:**
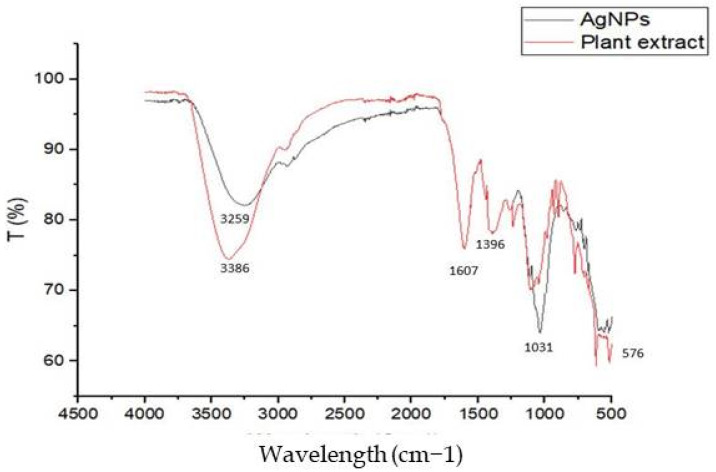
FTIR analysis of the green silver nanoparticles from *Achillea maritima* extract (with black color) and plant aqueous extract (with red color).

FTIR infrared spectroscopy analysis was performed to identify the bond linkages and the functional groups affiliated with the *Achillea maritima* extract treated with AgNO_3_. The FTIR spectrum shows that the identified groups play an important role in the reduction process of Ag ions and the formation of silver nanoparticles. The absorbance peak at 3259 cm^−1^ is attributed to the stretching vibration of O-H groups of carboxylic acids. The observed peaks at 1607 cm^−1^ and 1396 cm^−1^ correspond to N-H groups and N-aromatic groups, respectively [50,51]. The bands observed at 1031 and 576 cm^−1^ correspond to C-O groups of esters and C-N groups of amines and n-alkanes, respectively. The various functional groups detected may be good capping agents involved in AgNO_3_ reduction using *Achillea maritima* extract. The peaks at 1607 cm^−1^ and 1396 cm^−1^ correspond to NO_2_ asymmetric stretching; the same peaks are observed in plant extract and AgNPs, indicating that they are involved in the synthesis process of silver nanoparticles (Figure 5).

#### 3.1.4. Structural Properties

**Figure 6:**
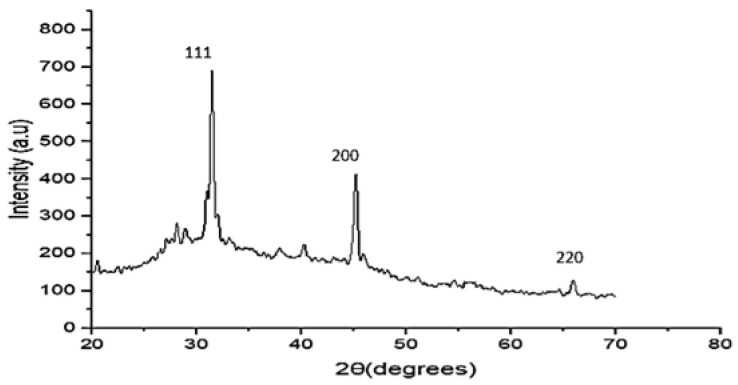
XRD pattern of silver nanoparticles biosynthesized using aqueous extract of *Achillea maritima* L. Peaks are attributed to diffraction from the (111), (200) and (220) planes of silver. Peaks of XRD pattern can be indexed as a face-centered cubic (fcc) structure (JCPDS, ref 4-0483).

The crystalline nature of nanoparticles was determined via X ray diffraction. XRD analysis shows the presence of diffraction peaks at, respectively, angles of 31.4°, 45.17° and 66.3°, which corresponds to major planes of (111), (200) and (220), respectively. These peaks are attributed to diffraction from the (111), (200) and (220) planes of silver. The peaks of the XRD pattern can be indexed as a face-centered cubic (fcc) structure (JCPDS, ref 4-0483). The detection of these planes reflects that the nanoparticles possess a face-centered cubic (fcc) structure specific to the spherical shape (Figure 6). By using the XRD pattern, based on the Scherrer mathematical equation, we calculate the size of the nanoparticles as follows:

d = K λ/ß cos θ

where d is the average crystalline size of the nanoparticles, k is the geometric factor equal to 0.9, λ = 1.5406 Å, and is the wavelength of Xray radiation, and ß is the angular FWHM (full-width at half maximum) of the XRD peak at a diffraction angle of θ [52]. The calculated size is 14.13 nm.

### 3.2. Antioxidant Activity

The results of free radical scavenging activity (DPPH) method show that the biosynthesized silver nanoparticles have significant antioxidant activity, with an IC50 value of 89.46 µg/mL (Table 1). The total antioxidant activity of the silver nanoparticles is about 1.88 mg AAE/gDM.

### 3.3. Antidiabetic Effect

**Figure 7:**
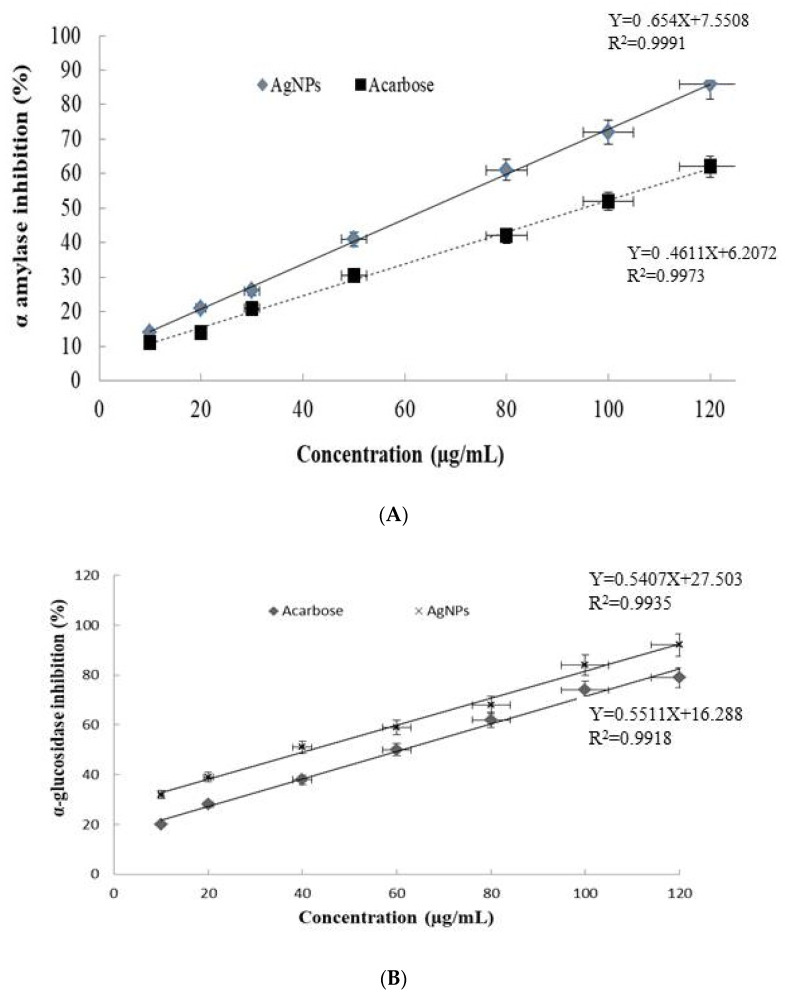
(**A**) α amylase’s inhibitory effect and (**B**) α glucosidase inhibition of silver nanoparticles from aqueous extract of *Achillea maritima* and acarbose (positive standard) used at 10 to 120 µg/mL. Error bars represent SE of the mean (*n* = 3), with *p* < 0.05.

Under in vitro conditions, the silver nanoparticles show significant α amylase and α glucosidase inhibition in a dose-dependent manner, higher than that shown by acarbose. The maximum α amylase inhibition is observed at the tested dose of 120 µg/mL for both the sample solution AgNPs, with 86%, and acarbose, with 62% (Figure 7A). The calculated IC50 values of the silver nanoparticles and acarbose are 64.9 and 94.83 µg/mL, respectively. For α glucosidase, the AgNPs show a marked inhibitory effect compared to acarbose; observed at 120 µg/mL with a percentage of inhibition of 92% and 79%, the IC50 values are of 41.6 and 61.17 µg/mL, respectively (Figure 7B).

### 3.4. Cytotoxic Effect of Silver Nanoparticles

**Figure 8:**
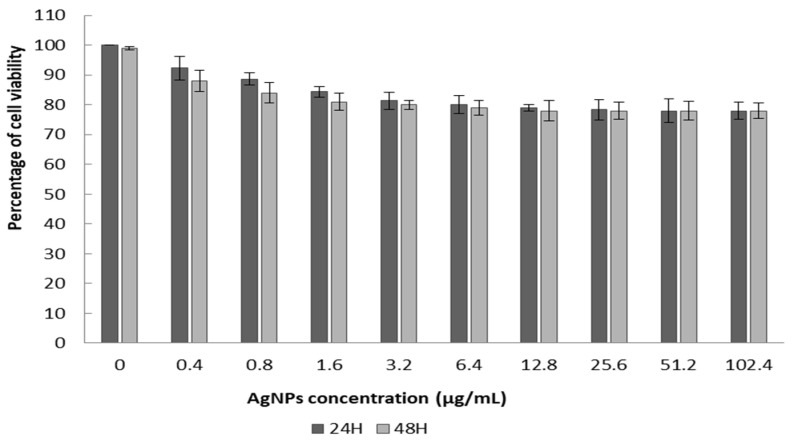
Cytotoxic potency of the AgNPs on cancer cell line after 24 h and 48 h exposure.

The percentage of cell culture viability when exposed to various AgNP concentrations (0.4 to 102.4 µg/mL) range from 79 to 100%. The IC50 values for the required concentration of tested AgNPs to inhibit 50% of cell viability were measured from the regression curves of percentage inhibition and AgNP concentration. After 24 h and 48 h, the IC50 values were 249.43 and 120.03 µg/mL, respectively (Figure 8). These results show that the silver nanoparticles exhibit no cytotoxic effect on colorectal LS174 cancer cells after 24 h and 72 h of incubation with silver nanoparticle solutions of 0.4 µg/mL to 102.4 µg/mL.

### 3.5. Antimicrobial Potential

#### 3.5.1. Agar Well Diffusion Method

Superior antibacterial activities were observed against both the bacteria species *E. coli* and *Salmonella typhi*, with zone inhibition diameters of 15.8 and 14.5 mm, respectively. The strongest antifungal activity was observed against *Candida albicans*, with 16.5 mm; this value exceeds that of the used standard Fluconazole 25 at 13.5 mm (Table 2).

#### 3.5.2. MIC, MBC and MFC Determinations

We determined MIC values ranging from 6.75 to 50 µg/mL related to the pathogen species. MBC/MIC and MFC/MIC ratios < 4 indicate bactericidal and fungicidal actions of the silver nanoparticles, as determined by Okou et al. [41] (Table 3).

#### 3.5.3. Silver Nanoparticles’ Effect on Bacterial Mechanism of Action

The obtained results show that silver nanoparticles have a great effect on the biofilm formation of the three tested bacterial species, with a percentage inhibition exceeding 62%. The highest percentage of lipopolysaccharide degradation is observed against *Escherchia coli*, with 65%, compared to *Salmonella typhi* and *Pseudomonas aeruginosa*, with values lower than 58% (Table 4).

**Figure 9:**
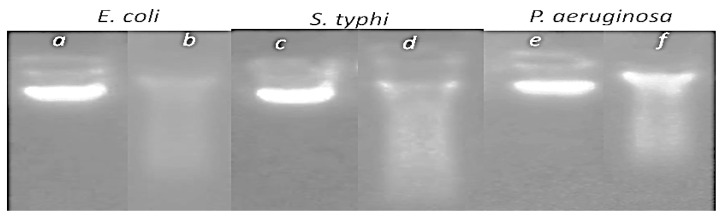
The genotoxic effect of silver nanoparticles, illustrated by gel electrophoresis of DNA genome of untreated bacterial strains (**a**,**c**,**e**) and those treated (**b**,**d**,**f**) with silver nanoparticles at 50 µg/mL, for *E. coli*, *S. typhi* and *P. aeruginosa*, respectively.

Moreover, the AgNPs damaged the DNA genomes of the bacterial strains. After 24 h of incubation of the bacterial cells, DNA extraction and migration via gel electrophoresis show that silver nanoparticles alter and damage DNA, as shown by an abnormal band (smaller DNA, break DNA) in the gel electrophoresis compared to intact DNA with one intensive band at the same position (Figure 9).

On the other hand, the silver nanoparticles were inactive against Gram-positive bacteria, which could be explained by the absence of its effect on the cell wall of Gram-positive bacteria since, in the current study, no lysozyme activity was detected against the tested cell walls of *Staphylococcus aureus*, *Bacillus cereus* and *Micrococcus luteus*. Overall, these findings suggest that silver nanoparticles specifically affect Gram-negative bacteria by acting on biofilm, lipopolysaccharides and DNA.

### 3.6. Silver Nanoparticles’ Effect on Candida albicans Growth and Virulence Factor 

**Figure 10:**
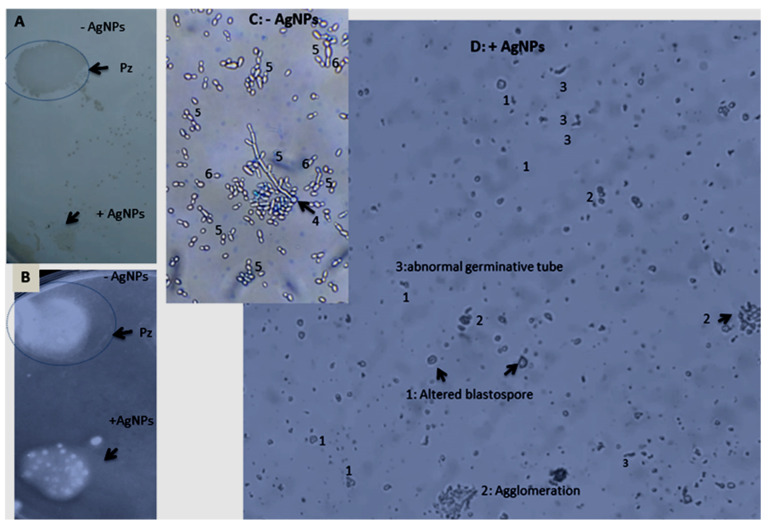
Comparison of production of extracellular hydrolytic enzymes (**A**) proteinase and (**B**) phospholipase and biofilm formation by *Candida albicans* species in the absence ((**C**):–AgNPs) and in the presence of AgNPs ((**D**): +AgNPs). The observation of zone precipitation around the *Candida* colony (Pz), indicating enzyme production. 1: altered blastospores, 2: agglomeration, 3: abnormal germinative tube (gr X1000).

The AgNPs moderately affected the cell growth of *Candida albicans* compared to the untreated culture, with a percentage of inhibition of 8.89% (Table 5). However, the AgNPs significantly limited extracellular enzyme production (proteinase and phospholipase), in addition to the marked inhibition of biofilm formation of *Candida albicans* observed following the application of AgNPs. The blockage of filamentous morphology and the alteration of the germinative tube and blastospores were also observed when *Candida albicans* was exposed to the AgNPs. The obtained results highlight the high limitation of the *Candida* virulence factor and germination (Figure 10).

## 4. Discussion

Several researchers from all over the word are focusing on nanomaterials, due to their eminent potential in various applications. Based on the huge amount of attention directed toward metallic nanoparticles, the first objective of the present work is the optimization of a simple and rapid method for the synthesis and the characterization of nanoparticles via spectroscopic analysis. Secondly, we describe their mechanism of action on bacterial and fungal cells to highlight their future use in the biomedical area. The current work highlights the application of UV-Visible and front-face fluorescence (FFF) spectroscopy to identify the shape and the size of nanoparticles. The results showed that the novel biosynthesized silver nanoparticles from *Achiella maritima* have a maximum peak at 420 nm in the UV-Visible spectrum, proving their spherical shape. The absorption band in the visible regions (390 nm to 500 nm) confirms the formation of surface plasmon resonance (SPR) by AgNPs, as mentioned in another study [17]. Moreover, a single SPR band in the range of 300 to 700 nm corresponds to the spherical shape of the nanoparticles, as previously reported [53]. The same peak at 420 nm was previously observed for AgNPs from de-oiled Zingiber officinalis and AgChem [54,55]. A single peak ranging from 420 nm to 470 nm was also observed for silver nanoparticles using leaf extract of *Aesculus hippocastanum* (horse chestnut) [56]. Peaks were observed at 390 nm and 420 nm from the aqueous and the ethanolic extracts of *Achillea millefolium* species, respectively [17]. The spherical shape of AgNPs from the aqueous extract of *Achillea millefolium* was also confirmed by a single peak [56,57].

It has been reported that the average sizes of AgNPs using the aqueous extract of *Achillea maritima* and *Achillea millefolium* were about 21 nm and 22.4 nm, respectively [17,58]. The same peak at 420 nm and a silver nanoparticle size of 24 ± 2 nm from algal extract have also been described [59]. In this study, the calculated size of 14.13 nm using XRD analysis was close to the size of 21.26 nm calculated via UV-Vis, so the silver nanoparticles’ size ranges from approximately 14.13 to 21.26 nm. The AgNPs from the *Achillea biebersteinii* flower extract showed a size of nearly 12 nm [60]. These results prove that the size of silver nanoparticles is related to the plant extract species and the experimental conditions [61].

It is well known that nanoparticles display important physical and chemical properties owing to their surface of particles and atoms. These properties give them various applications in materials technology, biomedicines and catalysis. Silver nanoparticles show surface plasmon absorption and plasmonic excited fluorescence [30,31]. Surface/interface interactions play a crucial role in their properties. In the current study, we propose FFFS to identify the emission spectra of silver nanoparticles synthesized using the aqueous extract of *Achiella maritima*. A prior work also examined the fluorescence characteristics of AgNPs under various excitants [27,28,29]. The component peaks seen in FFFS spectra are at the same position as those reported in the literature for similar compounds [30,31]. In the literature, the fluorescence emission of silver nanoparticles exited at 350 nm is reported to have the same peak of 470 nm [27].

In previous work, similar UV-Vis and fluorescence peaks at 422 nm and 470 nm, respectively, were also observed in silver nanoparticles [27]. Front-face fluorescence spectroscopy (FFF) is a non-destructive detection approach based on a spectroscopic analysis. It is a cheap method that does not use ionizing radiation and does not require the sample to be prepared in any way beforehand. In order to understand how silver nanoparticles behave in intricate biological contexts, it can be useful to examine them in their natural condition using this FFFS method. Fluorescence data obtained via FFFS can be converted into knowledge about the physical characteristics of nanoparticles using mathematical models and statistical analysis. These techniques have been widely exploited to characterize nanoparticles for use in numerous research applications [62,63,64].

Our FTIR analysis shows the richness with NO, proving the antioxidant and antimicrobial potential of the biosynthesized silver nanoparticles. The presence of C=O and C-O groups, in the plant extract confirms their critical roles as capping and reducing agents of AgNPs [65]. In previous work, chemical AgNPs were synthesized using AgNO_3_ as a silver precursor, aqueous NH_3_ as a pH adjustor and PVP as dispersant; here, these chemical agents (NH_3_ and PVP) were replaced by a plant extract, and the metal precursor (AgNO_3_) was involved in the Ag nanoparticle synthesis process [66]

New AgNPs from the aqueous extract of *Achillea maritima* exhibit stronger antioxidant potential (with DPPH radicals with an IC50 of 0.089 µg/mL) compared to the AgNPs from *Achillea millefolium* methanolic extract, with DPPH radicals with an IC50 of 7.03 µg/mL [17], and the AgNPs from *P. farcta* fruit extract with an IC50 of 0.7 mg/mL [67]. However, fewer antioxidant DPPH radicals, reflected by an IC50 of 67.1 µg/mL, are given by AgNPs using *Punica granatum* leaves [68]. The silver nanoparticles obtained using chemical methods also show antioxidant potential in the DPPH assay [69].

The present work mentions that the AgNPs show high alpha amylase and alpha glucosidase inhibition, with IC50 values of 64.9 and 41.6 µg/mL, respectively. A similar IC50 for α-amylase of 65.2 ug/mL was observed for the PGE AgNPs, as described in [69]. However, stronger activity was observed for our AgNPs against alpha glucosidase, with an IC50 of 41.6 µg/mL, compared to 53.8 µg/mL for PGE AgNPs [68]. In the literature, several silver nanoparticles exhibit an alpha amylase inhibitory effect, e.g., [70,71]. It is well known that alpha amylase and alpha glucosidase play crucial roles in carbohydrate metabolism. Their inhibition is one of the most remedial strategies for diabetes therapy [24,69]. Due to the undesirable side effects of the current diabetic drugs, such as acarbose and miglitol, the discovery of a new effective and natural antidiabetic drug is necessary. Silver nanoparticles from the medicinal plant present a safe and efficient antidiabetic agent for the treatment of diabetes. These findings confirm that the described AgNPs could be recommended as a good and safe antidiabetic agent for the treatment of diabetes.

Our silver nanoparticles do not exhibit any significant cytotoxicity on the tested cancer cell line, and there are no dose-dependent responses. When the IC50 values are >100 µg/mL, the substance in non-toxic [72], confirming the non-toxic properties of the AgNPs in accordance with ISO standards [73]. Similar results were observed for silver nanoparticles tested on peripheral blood mononuclear cells (PBMC) [74]. In the present study, we did not investigate inflammatory markers or cellular enzymes, but we did evaluate cell viability, which is an important approach to analyzing the cytotoxic effect of silver nanoparticles, as previously reported [75]. The toxicity of AgNPs depends on their size, shape and concentration and the nature of their synthesis. AgNPs synthesized from plant sources are less toxic in nature. However, researchers have reported the cytotoxicity of AgNPs in different cell lines, e.g., [5].

On the other hand, the biocompatibility of the new AgNPs, associated with their antidiabetic and antimicrobial potential, encourages their use in the formulation of novel pharmaceutical molecules and in various biomedical applications. In previous work, the chemical process of silver nanoparticle synthesis using PVP as a stabilizing agent and the oxidation process (with Oxide), and several optimization parameters, was undertaken to find moderately toxic AgNP [76]. In contrast, here, we propose safe AgNPs synthesized using the green method, with one simple step, that have antioxidant, antidiabetic and antimicrobial properties and a special non-toxic effect on a colorectal human cell line. These findings encourage the use of our natural AgNPs for oral administration as natural drugs.

In the current work, the described AgNPs possess stronger antibacterial action against Gram-negative than Gram-positive bacteria. In fact, it is well known that most AgNPs exhibit higher antibacterial activity against Gram-negative than Gram-positive bacteria. These findings may be explained by the thicker nature of the cell walls of Gram-positive bacteria, which act as a natural barrier against nanoparticle penetration. On the other hand, here, we have proven the alteration of lipopolysaccharides of Gram-negative bacteria with silver nanoparticles, due to the negative charge of LPS, which facilitates the adhesion of nanoparticles. In the literature, many works highlight that the main mechanism of action of nanoparticles involves electrostatic attraction between the negative charge of the microbial cell membrane and the positive or smaller charge of AgNPs [77]. Silver nanoparticles, after penetration into cells, can interact with lipid membranes and disrupt the cell wall content, such as proteins and DNA [78,79]. Similar results of a high antibacterial effect observed against a Gram-negative bacteria, *Escherchia coli*, were reported in [80]. In our work, we obtained AgNPs (at 50 µg/mL) and detected their mechanisms of action. In the future, we could test the effects of various AgNP doses on the mechanisms of action. Many works show that DNA damage is dose-dependent due to the degree of membrane alteration. Ag+ ions are able to interact with the nucleotides rather the phosphate groups of nucleic acids [52]. Moreover, the biosynthesized AgNPs from *A. maritima* show no cytotoxic risk in human cell CR cancer. In general, the green synthesis approach reduces toxicity concerns; in fact, the cytotoxic risk of AgNPs depends upon their sizes, shapes and density [80]. The damage to DNA was also evaluated for AgNP-treated *Pseudomonas aeruginosa*; the obtained results show lower PCR in treated culture and the strongest antibacterial effect on *P aeruginosa* compared to *Staphylococcus aureus* [81]. AgNPs could alter the adhesion of bacterial and fungal cells and prevent biofilm formation, which are significant pathogenicity determinants. In our previous works focused on AgNPs synthesized from other plants, we proved their anti-biofilm effect on bacterial and fungal cells [3,5]. In the current work, the biosynthesized AgNPs have a significant inhibitory effect on *Candida* virulence by affecting enzyme production and blockage of the filamentous form; these results are in accordance with previous works, e.g., [3,5,6,17]. The present work highlights a promising natural drug that can be used in the pharmaceutical area. On the other hand, the bioavailability, uptake and ADME-related properties of such drugs may present a barrier to their use [79]. For these reasons, to reach this goal, several studies report prediction models of the lipophilicity and aqueous solubility properties of molecules, such as Wieder et al. [80], who describe a novel graph-based neural network (GNN), which shows good performance in predicting the molecular properties of lipophilicity and solubility compared to other baseline models [80]. Wieder et al. [80] tested different training strategies and features to obtain more information compared to previous models’ performance via D-CIN, e.g., [81]. In light of these data, we suggest applying robust advanced deep learning methods such as GNNs for chemical property prediction of our AgNPs for the computational drug discovery pipeline.

## 5. Conclusions

There is a need for continuous research to enhance current antimicrobial drugs, so it is important to understand their microbial mechanism of action. Thus, the present work describes new silver nanoparticles using the simple green synthesis method, without any chemical agents. An immediate color change from yellow to brown and a surface plasmon resonance peak at 420 nm following UV-Vis spectroscopy confirmed AgNP formation. The biosynthesized AgNPs were spherical in shape and had an average size ranging from 14.13 to 21.26 nm. The AgNPs showed antioxidant potential following a DPPH assay, with IC50 = 0.089 µg/mL, and antidiabetic potential, as detected by alpha amylase and alpha glucosidase inhibition. The AgNPs indicated a significant antibacterial effect on Gram-negative bacteria due to their specific action on LPS, biofilm and DNA. Taken together, these results suggest that AgNPs prevent enzyme virulence and biofilm formation in *Candida albicans*, with no cytotoxic risk. The newly described silver nanoparticles present a promising natural antimicrobial agent to be added to the antimicrobial formation to treat bacterial and Candidiasis infections. Due to the lack of cytotoxic effect and the successful antidiabetic capacity of AgNPs from the extremophile plant *Achillea maritima*, we encourage using them in place of chemical AgNPs in oral drug administration. To achieve this goal, further investigations are proposed, such as the establishment of an advanced method for the molecular prediction of solubility and cell barrier interactions in animal models. In order to understand how AgNPs behave in intricate biological contexts, we propose FFFS to determine their physical characteristics in natural conditions, using mathematical models and statistical analysis.

## Figures and Tables

**Table 1:** Antioxidant potential and antiradical capacity of the silver nanoparticles compared to ascorbic acid.

DPPH Free Radical Scavenging Activity IC50 (µg/mL)	
AgNPs	89.46 ± 2.48 ^a^
Ascorbic acid	22.54 ± 0 ^b^
Total Antioxidant Activity (mg AAE/g DM)	
AgNPs	1.88 ± 0.01 ^a^
Ascorbic acid	0.80 ± 0.01 ^b^

Values are the means from triplicate experiments (means = 3). AAE: ascorbic acid equivalent, DM: dry matter. Different letters in the same column of each test show significant differences at *p* < 0.05, using the SNK test.

**Table 2:** Comparison of the diameter of zone inhibition obtained by the green synthesized silver nanoparticles from *Achillea maritima* subsp. *maritima* compared to the used standards.

Clinical Pathogens	AgNPs	Standards	
Bacteria		Ceftazidime CAZ30	Tobramycin
*Escherchia coli*	15.8 ^b^ ± 0	15 ^e^ ± 0	25 ^a^ ± 0
*Salmonella typhi*	14.5 ^c^ ± 0	26 ^b^ ± 0.5	18 ^d^ ± 0.5
*Pseudomonas aeruginosa*	12.5 ^d^	17 ^d^ ± 0	22 ^c^ ± 0.5
*Staphylococcus aureus*	0 ^e^	18 ^c^ ± 0	14.5 ^e^ ± 0
**Yeasts**		**Fluconazole 25**	**Amphotericin B**
*Candida albicans*	16.5 ^a^	13.5 ^f^ ± 0	23 ^b^ ± 0
*Candida tropicalis*	0 ^e^	35 ^a^ ± 0.5	22 ^c^ ± 0

Values are expressed in mm. Values with same letter in the same column are not significantly different according to SNK test at *p* > 0.05.

**Table 3:** Determinations of MIC, MBC and MFC values of AgNPs against the tested clinical pathogens. Values are expressed in µg/mL. The MBC/MIC ratio < 4 and the MFC/MIC ratio = 2.

Bacterial Strains	MIC	MBC	Ratio MBC/MIC
*Escherchia coli*	6.75 ^b^	12.5 ^b^	2 ^b^
*Pseudomonas aeruginosa*	6.75 ^b^	12.5 ^b^	2 ^b^
*Salmonella typhi*	6.75 ^b^	25 ^a^	4 ^a^
**Fungal Strains**	**MIC**	**MFC**	**Ratio MFC/MIC**
*Candida albicans*	12.5 ^a^	25 ^a^	2 ^b^

Values with same letter in the same column are not significantly different according to SNK test at *p* > 0.05.

**Table 4:** The inhibitory effect of silver nanoparticles on virulence factors of tested bacterial species.

Bacterial Species	Biofilm Eradication (I%)	Lipopolysaccharide Degradation (I%)	DNA Damage
*Pseudomonas aeruginosa*	79	47	+
*Escherchia coli*	62	65	+
*Salmonella typhi*	82	58	+

I (%): percentage of inhibition.

**Table 5:** Comparison of effects of silver nanoparticles on cell growth and virulence factor of untreated *Candida albicans* (-AgNPs) and that treated with AgNPs after 48 h of incubation at 37 °C.

Culture Conditions of *C. albicans*	Cell Growth DO600 nm, 48 h (%)	Biofilm Formation and Morphogenesis	Hydrolytic Enzyme Production (Pz in mm) ±SD	
			Lipase Pz	Proteinase Pz
**Untreated *C. albicans***	0.174 ± 0.04	+++ Morphogenesis change: germ tube; chlamydospores; pseudofilament	Pz = 0.6 ± 0	Pz = 0.54 ± 0.1
***C. albicans* treated with AgNPs at 50 µg/mL**	0.166 ± 0.03	(-) Altered blastospores (1), abnormal germinative tube (2), agglomeration (3)	Pz = 1 negative	Pz = 1 negative

Pz means zone of precipitation; Pz = 1 (negative); Pz: (0.9–0.99: +); Pz: (0.8–0.89 (++); Pz: (<0.7: ++++). Biofilm morphogenesis change (+++); absence of any morphogenesis change (-). SD: standard deviation.

## Data Availability

All data generated or analyzed during this study are included; any additional information is available from the corresponding author on reasonable request.

## Supplementary Materials

The following supporting information can be downloaded at: https://www.mdpi.com/article/10.3390/nano13131964/s1, Figure S1: examples of the antimicrobial agar well diffusion results. t: AgNPs (40 µL) or st: negative control (distilled water).
