# Supplementary material for: Green Synthesis and Characterization of Novel Silver Nanoparticles Using Achillea maritima subsp. maritima Aqueous Extract: Antioxidant and Antidiabetic Potential and Effect on Virulence Mechanisms of Bacterial and Fungal Pathogens
Source: Nanomaterials (Basel). 2023 Jun 28;13(13):1964. doi: 10.3390/nano13131964 (PMC10343889; doi:10.3390/nano13131964)
Supplement: Supplementary file 1 [file nanomaterials-13-01964-s001.zip › nanomaterials-2428100-supplementary.pdf]

nanomaterials

Green synthesis and characterization of novel silver nanoparticles using *Achillea maritima* subsp. *maritima* aqueous extract: antioxidant, antidiabetic potentialities and effect on virulence mechanisms of bacterial and fungal pathogens

Badiaa Essghaier[1,*], Hédia Hannachi[2], Rihem Nouir[3,4], Filomena Mottola[5] and Lucia Rocco[5*]

1 Biochemistry and Biotechnology laboratory LR01 ES05, Department of Biology, Faculty of Sciences of Tunis, University Tunis ELmanar 2092; badiaa.essghaier@fst.utm.tn.;
2 Laboratory of Vegetable Productivity and Environmental Constraint LR18ES04, Department of Biology, Faculty of Sciences, University Tunis El-Manar II,2092, Tunis, Tunisia; hedia.hannachi@fst.utm.tn.
3 Laboratoire de Spectroscopie Atomique, Moléculaire et Applications (LSAMA), Faculty of Sciences, Tunis El Manar University, 2092 Tunis, Tunisia,
4 Higher Institute of Medical Technologies of Tunis, Tunis El Manar University, 2092, Tunis, Tunisia, (R.M) rihem.nouir@gmail.com.
5 Department of Environmental, Biological and Pharmaceutical Sciences and Technologies (DiSTABiF) University of Campania "L. Vanvitelli", 81100 Caserta – Italy; filomena.mottola@unicampania.it (F.M.), lucia.rocco@unicampania.it (L.R).

* Correspondence: badiaa.essghaier@fst.utm.tn. (BE); lucia.rocco@unicampania.it (LR)

Figure S1 Antibacterial results by agar diffusion method

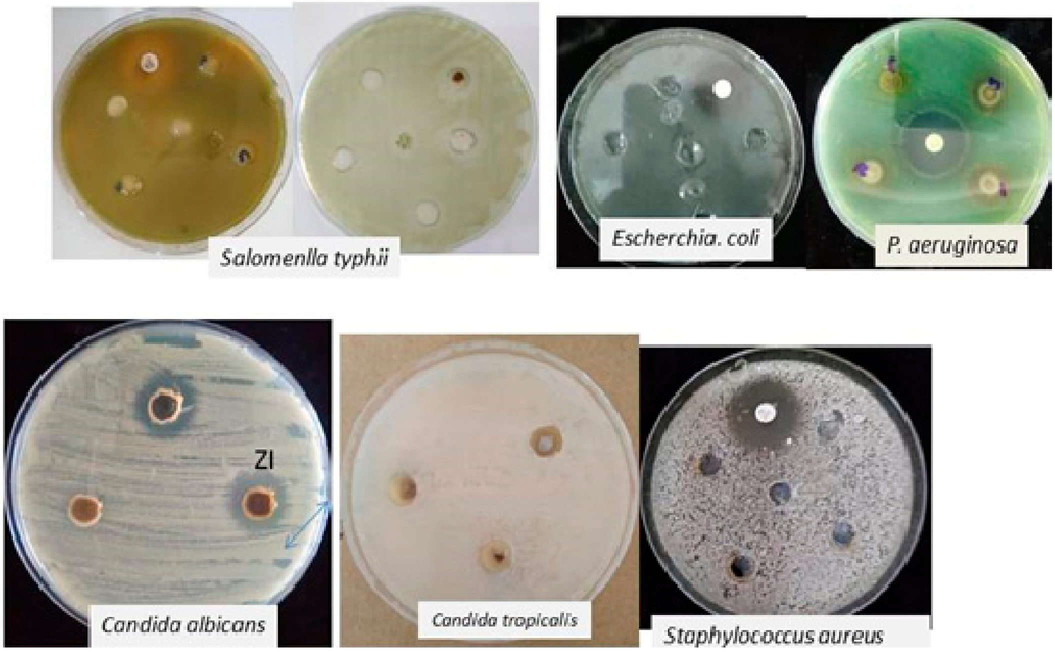

Figure S1: Antimicrobial agar well diffusion results. AgNPs (40µL) t: or st: negative control (water distilled)
